# Role of TRPV1 and TRPA1 Ion Channels in Inflammatory Bowel Diseases: Potential Therapeutic Targets?

**DOI:** 10.3390/ph12020048

**Published:** 2019-03-30

**Authors:** Kata Csekő, Bram Beckers, Daniel Keszthelyi, Zsuzsanna Helyes

**Affiliations:** 1 Department of Pharmacology and Pharmacotherapy, Medical School and Molecular Pharmacology Research Group, Szentágothai Research Centre, University of Pécs, H-7624 Pécs, Hungary; csekoe.kata@gmail.com; 2 Division of Gastroenterology-Hepatology, Department of Internal Medicine, Maastricht University Medical Center (MUMC+), 6202 AZ Maastricht, The Netherlands; ab.beckers@maastrichtuniversity.nl (B.B.); daniel.keszthelyi@maastrichtuniversity.nl (D.K.); 3 NUTRIM, School of Nutrition and Translational Research in Metabolism, Maastricht University, 6202 AZ Maastricht, The Netherlands; 4 PharmInVivo Ltd., H-7629 Pécs, Hungary

**Keywords:** IDB, Crohn’s disease, ulcerative colitis, TRPV1, TRPA1, human studies, animal studies, colitis models

## Abstract

Inflammatory bowel diseases (IBD) have long been recognized to be accompanied by pain resulting in high morbidity. Transient receptor potential vanilloid 1 (TRPV1) and ankyrin 1 (TRPA1) ion channels located predominantly on the capsaicin-sensitive sensory neurons play a complex role in hyperalgesia and neurogenic inflammation. This review provides an overview of their expression and role in intestinal inflammation, in particular colitis, that appears to be virtually inconsistent based on the thorough investigations of the last twenty years. However, preclinical results with pharmacological interventions, as well as scarcely available human studies, more convincingly point out the potential therapeutic value of TRPV1 and TRPA1 antagonists in colitis and visceral hypersensitivity providing future therapeutical perspectives through a complex, unique mechanism of action for drug development in IBD.

## 1. Introduction

Inflammatory bowel disease (IBD) is a group of chronic relapsing and remitting inflammatory disorders of the bowel [1,2]. The two major groups of IBD are Crohn’s disease (CD) and ulcerative colitis (UC), each having its own disease characteristics. The cardinal symptom in both disorders however, is abdominal pain, which often causes significant morbidity. Transient receptor potential vanilloid 1 (TRPV1) and ankyrin 1 (TRPA1) ion channels located predominantly on the capsaicin-sensitive sensory neurons play a complex role in hyperalgesia and neurogenic inflammation, and although their role in colitis is seemingly contradictory, there is a growing evidence on their involvement in IBD. The aim of this review is therefore to provide an overview of their expression and role in intestinal inflammation, in particular colitis.

CD is characterized by lesions affecting the entire gastrointestinal tract, UC per definition is limited to the colon. In addition, whereas inflammation is usually confined to the mucosa in UC, it often extends beyond the muscular layers in CD (i.e., transmural inflammation). Microscopically, one can sometimes identify characteristic differences, with non-caseating granulomas present in CD and crypt abscesses in UC. Even clinical differences exist, as bloody stools are commonly seen in UC but far less often in CD. On the other hand, perianal disease (including fistulas and perianal abscesses) is more suggestive for CD than UC. Other symptoms are encountered in both UC and CD, including malaise, fatigue, diarrhoea and loss of appetite.

As mentioned above, disease activity varies during the course of the disorder, meaning that both the extent of inflammation and patient symptoms change over time. In order to monitor disease activity, both clinical and biochemical assessments are used, as well as endoscopic follow-up. Currently commonly used biomarker is fecal calprotectin (a non-invasive inflammatory marker that correlates well with disease activity in both UC and CD) [3]. No other biomarkers have yet been established as an instrument to monitor disease activity in clinical practice.

Although biochemical/endoscopic signs of disease activity are important to monitor, it should be noted that IBD patients often report symptoms during clinical and biochemical remission. These symptoms may include abdominal pain, bloating, or a feeling of incomplete rectal evacuation, accompanied by a disturbed bowel pattern. Since these symptoms are compatible with irritable bowel syndrome, they are often referred to as “IBS-like”. In clinical practice, treatment of these IBS-like symptoms in IBD patients remains particularly challenging. Increasing understanding of the biological and molecular background of these phenomena can therefore potentially contribute to the development of novel treatment paradigms.

## 2. Inflammatory Bowel Diseases (IBD) and Related Pain

Inflammatory states have long been recognized to be accompanied by pain, as captured by the early Latin definition of inflammation (*calor, rubor, tumor* and *dolor*) [4]. In line with this definition, abdominal pain is a cardinal symptom of IBD [5]. Inflammation is likely the primary cause of abdominal pain in active IBD and one of the main suspects in the pathophysiology of ongoing complaints during remission, although the latter is still a subject of discussion [6]. Over 30% of UC patients in remission and almost double this percentage in quiescent CD patients have IBS-like symptoms [7]. On the basis of pain symptoms, one could expect visceral hypersensitivity to be present in active IBD and in symptomatic quiescent IBD patients. Already in the late seventies, Farthing and colleagues studied sensory responses to rectal balloon distensions in patients with ulcerative colitis, as compared to healthy controls [8]. Not surprisingly, it was demonstrated that patients with active ulcerative colitis tolerated a far smaller balloon volume than healthy controls. Similar findings were later reported by two other research groups [9,10]. Moreover, Rao and co-workers showed that hyperalgesia largely subsided upon remission. Finally, Hoboken and colleagues observed rectal hypersensitivity in UC patients in remission that reported IBS-like symptoms. However, these IBS-like symptoms do not appear to be associated with initial extent of the disease [7]. Moreover, no differences have been found in fecal calprotectin between UC patients in remission with or without IBS-like symptoms [11,12]. It is therefore more likely that the acute inflammatory phase induced lasting changes in visceral nociception in these patients, rather than low-grade mucosal inflammation causing ongoing symptoms during remission. 

Paradoxically, two more recent studies reported similar or even higher rectal discomfort thresholds in IBD patients (either active disease or in remission) as compared to healthy controls [13,14]. However, important differences with the above mentioned studies should be kept in mind when interpreting these results. First, Bernstein and co-workers studied CD patients with isolated ileal involvement only, with balloon distensions consequently being applied to non-inflamed tissue. The observed higher rectal discomfort thresholds in CD patients during a ramp distension protocol would therefore more likely represent a form of central compensation due to a chronic inflammatory state and subsequent nociceptive signaling, rather than an effect at the peripheral level. The same research group conducted a similar study in UC patients. Using a threshold tracking paradigm, which is considered a non-biased distension protocol as the direction of each step depends on the patient’s response during the previous distension [15], it was demonstrated that UC patients had similar rectal discomfort thresholds as healthy volunteers. It should be noted, however, that UC patients in this study were either in remission or were reported to have only mild disease activity, with most patients being asymptomatic. Results therefore do not necessarily contradict the earlier balloon distension studies, in which patients often had significant disease activity. 

## 3. Transient Receptor Potential Vanilloid 1 and Ankyrin 1 Pain Sensing Ion Channels

Transient receptor potential (TRP) ion channels comprise more than 30 structurally related ion channels, divided into the TRPC (Canonical), the TRPV (Vanilloid), the TRPM (Melastatin), the TRPP (Polycystin), the TRPML (Mucolipin), the TRPA (Ankyrin) and the TRPN (NOMPC) subfamilies based on their sequence homology [16]. Most of them are non-selective cation channels, however, they exhibit differences in permeability and selectivity [17]. These ion channels are tetramers composed of six transmembrane domains, with a pore formed by the hydrophobic region between the fifth and sixth segments. They can assemble as homo- or heterotetramers to form functional units [18]. The physiological role of TRP channels ranges from store-operated calcium channels to thermo-, mechano- and chemosensors. 

The most investigated members of the family in relation to gastrointestinal inflammation include vanilloid 1 (TRPV1), ankyrin 1 (TRPA1). They are located predominantly on the capsaicin-sensitive sensory neurons, but several non-neural expressions have recently been described that drew great attention to this research area [19]. In general, they are activated by a variety of exogenous chemicals and endogenous mediators making them important regulatory structures in inflammatory and pain processes. Here we focus on TRPV1 and TRPA1, since there are many experimental and clinical results describing their expression and importance in the gastrointestinal tract most importantly in the colon. 

TRPV1 and TRPA1 are polymodal nociceptors playing an important role in thermo- mechanical- and chemo-sensation, and play a complex role in hyperalgesia and neurogenic inflammation. Their endogenous activators are often produced during inflammation, e.g., lipoxygenase products, the acidified pH of the inflamed tissue, and the gastrointestinal mucosa is frequently exposed to their exogenous agonists, such as capsaicin, allyl isothiocyanate, allicin etc. ingested by food. Furthermore, TRPV1 and TRPA1 are capable of functional interaction, such as heterologous desensitization [20], since the majority of TRPA1 expressing nerve fibers co-express TRPV1 [21]. Both ion channels can be sensitized by a variety of other mechanisms, such as prostaglandins, bradykinin and proteases, e.g., cathepsin expressed by immune cells, via the protease-activated receptor 2 (PAR2) present on both capsaicin-sensitive nerve endings and the immune cells themselves [22,23]. 

### TRPV1 and TRPA1 in IBD Patients

Putative evidence points toward sensitization and even activation of TRPV1 by various inflammatory mediators, as indicated by the multiplicity of animal studies reported below. In vitro studies using human embryonic kidney cells (HEK293 cells) transfected with rat TRPV1 cDNA have reported TRPV1 sensitization by various mediators of inflammation as well, including prostaglandin E2 and prostaglandin I2, bradykinin, nerve growth factor and the chemokine CCL3 [24,25,26,27]. Moreover, in a study with rectal biopsy material from healthy volunteers, pre-incubation with histamine was shown to potentiate TRPV1 responses. Inflammation associated tissue acidification would furthermore appear to be an obvious route for TRPV1 activation [28]. Indeed, intradermal and intramuscular low pH injections in healthy volunteers were shown to elicit moderate pain responses, which were potentiated by the injection of prostaglandin E2 [29]. Similarly, Jones and colleagues demonstrated that topical application of capsaicin potentiated pain responses to iontophoresis of protons [30]. It should be noted, however, that desensitization after repeated topical capsaicin application did not reduce acid-induced pain responses, suggesting that other receptors are involved as well. Nonetheless sufficient data indicates TRPV1 is sensitized in inflammatory conditions, thus potentially resulting in hyperalgesia and abdominal pain in IBD. 

Although TRPV1 appears to be sensitized by inflammatory mediators, studies on the expression of TRPV1 in inflamed human intestine, and in particular colon tissue have yielded contradictory results (Table 1 and Table 2). Yiangou and co-workers previously investigated TRPV1 immunoreactivity in colonic tissue samples from IBD patients who underwent a colectomy due to refractory disease, using tissue samples obtained from resections due to non-obstructing carcinoma as controls [31]. It was demonstrated that TRPV1 immunoreactivity was greatly increased in the colonic nerve fibers of patients with IBD as compared to controls. These findings were recently corroborated by a study with samples from 60 IBD patients (30 patients with UC and 30 patients with CD) [32]. Whereas Yiangou observed increased expression in the submucosa only, Luo and colleagues reported increased TRPV1 expression in both the mucosa and infiltrating inflammatory cells. On the other hand, we observed decreased levels of TRPV1 mRNA in biopsy material from patients with active and inactive CD and UC as compared to healthy controls [33]. In line with our findings, Rizopoulos and colleagues very recently reported decreased TRPV1 expression in mucosal biopsy material from UC patients, as compared to colonic resections from non-IBD patients [34]. Importantly, TRPV1 expression does not appear to be correlated with disease activity, arguing against a role for the extent of inflammation. Regardless of the direction of regulation, inflammation induced changes in TRPV1 expression are likely reversible. Akbar and co-workers found no differences in TRPV1-immunoreactivity in rectosigmoid biopsies when comparing samples from asymptomatic quiescent IBD patients and healthy volunteers, but did find increased TRPV1 expression in quiescent IBD patients with abdominal pain [35]. Similarly, we did not observe significant differences in TRPV1 transcription in sigmoid colonic mucosal samples from (primarily asymptomatic) quiescent UC patients, as compared to healthy controls [36].

There are few data regarding the expression and function of TRPA1 in IBD patients. However, results appear to be less contradictory than with TRPV1 (Table 1 and Table 2). In our study with biopsy material from patients with active and inactive CD and UC, we found a significant TRPA1 mRNA upregulation. Similarly, Bertin and co-workers found TRPA1 to be upregulated in patients with active UC and CD, although the difference was non-significant because of small sample size (*n* = 13). Triple immunofluorescence staining for TRPV1, TRPA1 and CD4 demonstrated that infiltrating CD4+ T cells were also positive for TRPV1 and TRPA1 [37]. Moreover, a significantly higher number of these cells was found in the colonic tissue samples of both UC and CD patients. Two other studies also reported increased TRPA1 expression in stenotic regions in the colon of CD patients, in samples obtained surgically and endoscopically. These studies suggested TRPA1 to be anti-fibrotic. Using a culture medium containing normal human intestinal myofibroblasts (InMyoFibs), it was demonstrated that adding type I collagen to the medium enhanced TRPA1 expression [38]. When fibrosis was elicited by transforming growth factor β1, knockdown of TRPA1 with siRNA resulted in enhanced fibrogenic effects [38]. 

## 4. Animal Models of IBD

Unfortunately ideal IBD models with real translational value do not exist in animals, because they cannot completely mimic the complexity of the multifactorial psychosomatic disease. Moreover, as colitis models usually involve short-term administration of an irritating substance, these rather represent acute inflammation. This is a significant limitation of these models in the context of their representation of IBD, which is a chronic disease. In addition to the duration of administration, the type of irritating substance being used determines the characteristics of the model. The fact that model specifics can influence the results should be taken into consideration in their interpretation. We have to rely on well-established and characterized mechanism models exhibiting most autoimmune and inflammatory components of the human disease [40]. Since human studies revealed a potential role of TRPA1 and TRPV1 receptors in the pathogenesis of IBD, but only expression changes could be detected in the human samples, preclinical tests are essential to have a better insight into the pathogenesis of IBD, investigate functional alterations including the role of these ion channels, as well as perform pharmacological interventions. 

Besides chemical induction and bacterial infection (with *Salmonella typhimurium* and *Salmonella dublin* or invasive-adherent *Escherichia coli*) several transgenic and knockout strains have been developed in order to investigate the specific pathophysiologic alterations in IBD. 

Administration of 1%–5% dextran sulfate sodium (DSS) in the drinking water of animals is a widely used method of chemically-induced colitis by disrupting the tight junctions between the intestinal epithelial cells and inducing inflammation through exposing the lamina propria to bacterial and other toxins, infective agents and antigens [41]. The consequent inflammatory cascade with a characteristic symptomatology (bloody diarrhea, weight loss and histopathology with inflammation limited to the mucosa, as well as cytokine profile) is considered to model UC with several limitations such as great variability between the experimental paradigms including concentration, molecular weight and sulphate content of DSS, intestinal flora, strain differences, administration protocol and timing, as well as the endpoints [42].

Further UC-related rodent models include the intrarectal administration of oxazolone and acetic acid. Oxazolone induces a characteristic T helper 2 (Th2) predominant immune response associated with epithelial cell loss in the colon, acetic acid administration evokes direct chemical damage (erosions, ulcerations accompanied by crypt abnormalities) in the distal colon. 

In contrast to these UC-like models, trinitrobenzene or dinitrobenzene sulfonic acid (TNBS/DNBS) colitis is associated with profound transmural infiltration of inflammatory cells and Th1-mediated immune response, thus resembling more to CD [43]. 

For the investigation of T-cell-mediated pathogenesis of colitis, IL-7 overexpressing and T-cell receptor α chain (*TCRα*) deficient (knockout: KO) mice are used in acute and chronic models, respectively, associated with neutrophilic and lymphocytic infiltration [44,45]. Other mouse strains developing spontaneous colitis include Wiskott-Aldrich syndrome protein (*WASP*) KO mice with characteristically elevated levels of Th2 cytokines [46]. Meanwhile, 25% of mice lacking the multidrug resistance 1a gene (*Mdr1α* KO) also show similar symptoms due to a decreased production of IL-10 and functional Treg cells [47]. Furthermore, *IL-2* KO, as well as guanine nucleotide-binding protein subunit α-2 (*Gαi2*) KO mice exhibit UC-like phenotype with crypt abscess formations and ulcerations [48,49].

### 4.1. Expression of TRPV1 and TRPA1 in Animal Colon

In the gastrointestinal tract TRPV1 is often co-expressed with TRPA1 in capsaicin-sensitive extrinsic sensory nerves, especially in the primary sensory neurons of the dorsal root ganglia. The density of these TRPV1 positive fibers increase from proximal to distal regions of the colon in mice [50]. Furthermore, during DSS colitis the proportion of DRG neurons expressing TRPV1, and their relative TRPV1 mRNA levels increase with a subsequently elevated release of sensory neuropeptides, such as calcitonin gene-related peptide (CGRP) and substance P (SP) [51]. Although the role of TRP-expressing afferents in inflammation is undisputable, there is growing evidence on the expression of TRPV1 and TRPA1 in intrinsic sensory neurons of the myenteric and submucosal plexuses [33,50,52,53] as well as on the surface epithelial cells of colonic mucosa [33,53,54]. The importance of sensory-immune interactions in colonic inflammation is also supported by the expression of TRPV1 and TRPA1 on inflammatory cells like mucosal macrophages, as well as CD4+ T cells [33,37,55] (Table 3 and Table 4).

### 4.2. Role of TRP Channels in Animal Models of Colitis

The role of TRP channels, in particular TRPV1 and TRPA1 is virtually contradictory in the pathogenesis of IBD. Several studies have been focused on elucidating the mechanism by which these channels might mediate pro-inflammatory and/or anti-inflammatory effects (Table 5).

Goso and co-workers provided the first evidence for a protective role of TRPV1-expressing peptidergic sensory nerves via the release of the protective neurotransmitter CGRP upon acute co-administration of capsaicin in a TNBS-induced colitis model [60]. Administration of TRPV1 agonists, resiniferatoxin (RTX) or high dose capsaicin, induces a sustained functional denervation of TRPV1-expressing extrinsic neurons, thus it provides a method in animal models for the investigation of these sensory afferents and the released neurotransmitters. The results of this chemical desensitization are not coherent, since pro-inflammatory and protective roles have also been described. Neonatal capsaicin desensitization, as well as the administration of the TRPV1 antagonist capsazepine have been reported to significantly attenuate macroscopic damage score, myeloperoxidase (MPO) activity increase (peroxidase enzyme released from neutrophil granulocytes in the inflamed tissues) and inflammatory histopathological alterations compared to normal DSS-treated rats attributing the colitogenic effect to SP released from the nerve terminals of TRPV1-expressing sensory fibers [56]. Meanwhile, Utsumi and co-workers found opposing results in the same model after adult treatment by high doses of capsaicin, which exacerbated colitis and reduced the inflammation-induced upregulation of both SP- and CGRP-positive fibers [64]. However, they described that TRPV1 and TRPA1 gene deletion decreased colitis severity and the upregulation of SP-positive nerve fibers without influencing protective CGRP-positive nerves. Similarly, neonatal capsaicin denervation resulted in more severe colitis in the oxazolon-induced model, but exacerbation was not accompanied by changes in the expression and distribution of CGRP- and SP-immunoreactive nerves in the colon [68]. These virtually contradictory pro- and anti-inflammatory effects of neuropeptides released from the TRPV1/A1-expressing fibers during chemically-induced colitis were further investigated in the TNBS model, where abrogated CGRP release in the isolated colon preparations and dorsal root ganglia were observed in *Trpa1*, but not in *Trpv1* gene-deficient mice. They showed that this mechanism is mediated via the sustained sensitization of TRPA1 by TNBS covalently binding to the cysteine and lysine residues in the cytoplasmic N-terminus of the receptor protein. TNBS induces similar severe acute colitis in wildtype and *Trpv1^−/−^*, but reduced inflammation in *Trpa1*^−/−^ mice or wildtype animals treated with the TRPA1 antagonist HC-030031. Sensory denervation, as well as *SP* gene-deletion abolished both TNBS and DSS-induced colitis, while in *CGRP*-deficient mice TNBS induced a more severe colitis further supporting the opposing actions of the sensory neuropeptides released from the same nerve terminals [51,66]. *Trpv1*-deficiency did not affect disease severity, only prevented chronic pain development during the recovery phase of DSS-induced colitis [67]. However, this result was challenged by other studies demonstrating the pathogenic role of TRPV1 by gene-deleted mice exhibiting less severe DSS-induced colitis, concluding that inflammatory mediators activate the TRPV1 receptor and induce neurogenic inflammatory components by releasing SP, neurotensin, vasoactive intestinal polypeptide and galanin [64,69]. Meanwhile, Massa and co-workers found more severe DNBS-induced colitis in *Trpv1^−/−^* mice, suggesting a protective role of TRPV1 [65]. Bertin and co-workers proposed non-neuronal TRPV1 and TRPA1-mediated proinflammatory mechanisms in colitis. They showed that both channels are present on mouse and human CD4+ T cells and play an important regulatory role in their activation and the production of proinflammatory cytokines, such as interferon- γ (IFN-γ), interleukin-2 (IL-2), IL-10 and tumor necrosis factor α (TNFα). In a spontaneous *IL10^−/−^* colitis model both genetic deletion and pharmacologic inhibition of TRPV1 resulted in attenuated inflammation. They provided clear experimental evidence in a T cell adoptive transfer model that TRPV1-expressing CD4+ T cells are involved in colitis pathogenesis [55]. In the same experimental paradigm TRPA1 was described to exert protective actions by restraining TRPV1 activity on these immune cells, thus controlling their activation and inflammatory functions [37]. The protective role of TRPA1 was also supported by TRPA1-mediated downregulation of proinflammatory neuropeptides SP, neurokinins A, B (NKA, NKB) and NK1 receptor, as well as cytokines and chemokines like TNFα, IL-1β, monokine induced by gamma interferon (MIG) and monocyte chemotactic protein-1 (MCP-1) [33].

Pharmacological interventions with curcumin had anti-inflammatory and anti-hyperalgesic effects in colitis models [61,62]. Although in these studies curcumin was interpreted and discussed as a TRPV1 agonist, it is important to note that curcumin is a non-selective compound having a typical pleiotropic effect including direct antioxidant activity, anticancer and antimicrobial properties mediated by a wide range of targets, even the TRPA1 receptor [70,71,72]. Considering that TRPA1 is almost exclusively expressed in TRPV1-positive neurons and both channels are known to interact [20,21], cross-desensitization could have a role in the actions of curcumin. Furthermore, curcumin was also described as a TRPV1 antagonist, because it inhibited capsaicin-evoked potentials [73]. In a clinical study, curcumin was reported to significantly reduce relapse rate in UC patients not via the activation, but the inhibition of TRPV1 either directly or by ways of cross-desensitization of TRPA1 [74]. However, we should be cautious when drawing conclusions regarding TRPV1 involvement based on the results of curcumin administration. 

Cannabinoids have also shown beneficial effects in animal colitis models [65,75]. Changes in the endocannabinoid system during intestinal inflammation have been described and TRPV1-associated effects could be involved in the anti-inflammatory effects of cannabinoids [75,76,77]. More than two-fold increase of anandamide also acting as a TRPV1 agonist was described in the human UC biopsy samples [75]. A single oral dose of the endocannabinoid palmitoylethanolamide (PEA) was shown to increase 2-arachidonoylglycerol (2-AG) blood levels in human volunteers [78]. In HEK-293 cells transfected with human recombinant TRPV1, PEA significantly enhanced 2-AG induced activation and desensitization of TRPV1. It was therefore speculated that 2-AG is responsible for the protective effect of PEA during an induced inflammatory response [79]. In a study using colonic explants of six quiescent IBD patients, it was demonstrated that treatment with PEA and cannabidiol (CBD) suppressed secretion of inflammatory mediators in explants exposed to inflammatory cytokines that was counteracted by the TRPV1 antagonist [77]. 

Other TRPV1 and TRPA1 antagonists also showed mainly protective actions. TRPV1 blockade by the non-selective antagonist capsazepine, JNJ 10185734, BCTC and SB366791 in various models of colitis exerted anti-inflammatory actions supporting the pathogenic role of TRPV1 in experimental IBD [55,56,57,58,59]. Moreover, both intraperitoneal and intrathecal administrations of TRPV1 and TRPA1 antagonists exerted analgesic actions in rat colitis models highlighting central nervous system mechanisms [59].

## 5. Conclusions, Drug Developmental Perspectives

**Figure 1:**
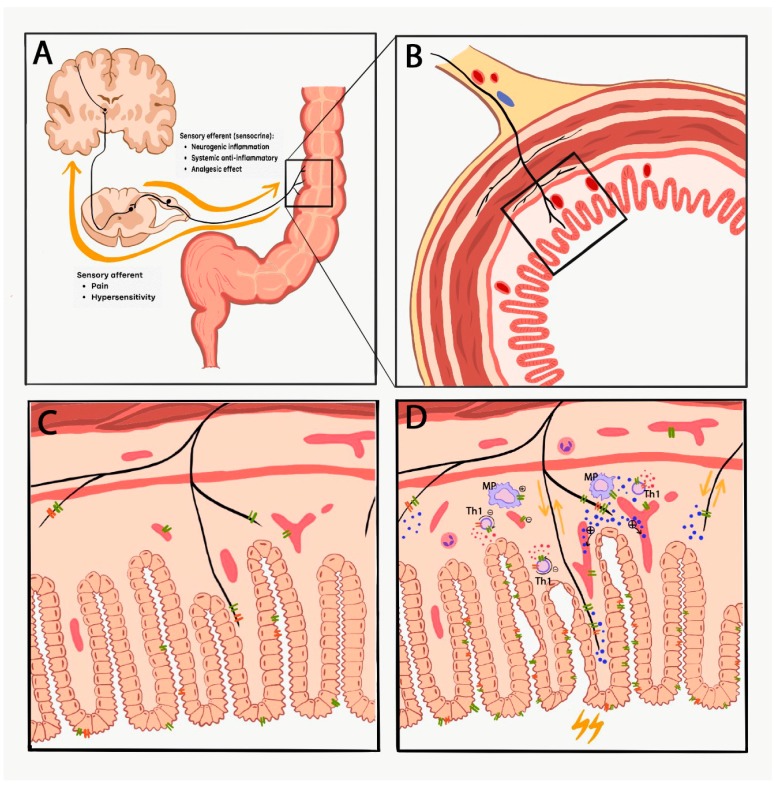
The complex interactions of TRPV1 and TRPA1 and their virtually contradictory role in colitis. Panel A demonstrates the afferent and efferent (sensocrine) functions (yellow arrows) of the capsaicin-sensitive sensory nerve fibers. Panel C (without inflammation) and D (during inflammation) depict an enlarged schematic section of the colon cross section (panel B) focusing on the expression and interaction of TRPV1 (green double lines) and TRPA1 (orange double lines) in the colon mucosa. Neurogenic inflammation is mediated via the release of SP and CGRP (blue dots represent neurotransmitters, such as SP, CGRP and somatostatin), resulting in increased vascular permeability, plasma protein extravasation and inflammatory cell activation. Meanwhile, anti-inflammatory sensory neuropeptides, such as somatostatin and opioid peptides released simultaneously from the same nerve ending exert anti-inflammatory and analgesic actions both locally and systemically through getting into the circulation. Furthermore, these ion channels on vascular smooth muscle and inflammatory cell such as macrophages (MP) and T helper cells (Th1) mediate both pro- (+) and anti-inflammatory (-) effects by regulating the release of cytokines (IFN-γ, IL-2, IL-10, TNFα are represented as red dots).

TRPV1 and TRPA1 expression, and experimental data regarding its role in colitis appears to be virtually inconsistent. Activation of these receptors on sensory nerve terminals mediates neurogenic inflammation via the release of SP and CGRP, resulting in increased vascular permeability, plasma protein extravasation and inflammatory cell activation. Meanwhile, anti-inflammatory sensory neuropeptides, such as somatostatin and opioid peptides released simultaneously from the same nerve ending exert anti-inflammatory and analgesic actions both locally and systemically through getting into the circulation. Furthermore, these ion channels on vascular smooth muscle and inflammatory cells such as macrophages and T helper cells mediate both pro- and anti-inflammatory functions. Therefore, the overall role of TRPV1 and TRPA1 in experimental colitis is dependent on (1) the diversity of the expression of these ion channels on sensory nerves, immune cells, epithelial cells and vascular smooth muscle cells [19], (2) the consequent activation-induced release of broad range of pro- and anti-inflammatory mediators including sensory neuropeptides and cytokines exerting divergent mechanisms, (3) the complex interactions of the co-expressed TRPV1 and TRPA1 receptors (Figure 1), (4) differences of the experimental models, protocols and paradigms (species, strain, concentration and composition of the chemicals, duration, intensity, complex mechanisms of the injury), as well as several limitations of the models [42]. 

However, preclinical results with pharmacological interventions, as well as scarcely available human studies, more convincingly point out the potential therapeutic value of TRPV1 and TRPA1 antagonists in colitis and visceral hypersensitivity providing future therapeutical perspectives for small molecule candidates. The first generation TRPV1 compounds thoroughly investigated in a broad range of clinical trials as novel analgesic and anti-inflammmatory drugs interfered with thermoregulation, elicited severe hyperthermia [80,81] and raised heat pain thresholds with consequently increased burn risk [82,83]. Therefore, they could not be registered for the clinical practice. Second generation new drugs with different inhibition sites on the TRPV1 and/or TRPA1 antagonists without the hyperthermic side effect could provide solutions to these problems [84]. Their clinical efficacies are currently intensively investigated, but they could open new perspectives through a complex, unique mechanism of action for drug development in IBD [85,86]. 

## Figures and Tables

**Table 1:** Alterations in TRPV1 and TRPA1 transcription/expression in Crohn’s disease (CD) patients with fold-changes where available. (IHC: immunohistochemistry, IF: immunofluorescence).

Disease Activity	Ion Channel	Sampling Method/Location	Methods	Results; Number of Patients	Relation to Abdominal Complaints and/or Disease severity	Ref
Active CD	TRPV1	Resection (colectomy)	IHC (computerized image analysis)	upregulated in submucosa; *n* = 6	Not reported	[31]
		Colon biopsy–affected and non-affected regions	IHC (computerized image analysis)	upregulated in mucosa and infiltrating inflammatory cells; *n* = 30	No significant correlation between disease severity and TRPV1 expression	[32]
		Distal colon biopsy	IHC, qPCR	downregulated mRNA *n* = not reported	Not reported	[33]
		Colon biopsy	IF	downregulated mRNA *n* = 6	Not reported	[37]
	TRPA1	Distal colon biopsy	IHC, qPCR	upregulated mRNA *n* = not reported	Not reported	[33]
		Colon biopsy	IF	upregulated mRNA *n* = 7	Not reported	[37]
CD in remission	TRPV1	Distal colon biopsy	IHC, qPCR	downregulated mRNA *n* = not reported	Not reported	[33]
		Rectosigmoid biopsy	IHC	upregulated in symptomatic quiescent patients; 3.9-fold increase in median number of TRPV1-immunoreactive fibers (CD and UC combined) *n* = 9	Significant correlation between TRPV1 expression and abdominal pain score	[35]
CD – disease activity unknown	TRPA1	Surgical samples of fibrotic regions (colon)	IHC	Denser immunoreactivity in mucosal and submucosal layers *n* = 3	Not reported	[38]
		Biopsy from fibrotic regions (colon)	IHC, RT-PCR	upregulated mRNA and protein levels *n* = 8	Not reported	[39]

**Table 2:** Alterations in TRPV1 and TRPA1 transcription/expression in ulcerative colitis (UC) patients with fold-changes where available.

Disease Activity	Ion Channel	Sampling Method/Location	Methods	Results; Number of Patients	Relation to Abdominal Complaints and/or Disease Severity	Ref
Active UC	TRPV1	Resection (colectomy)	IHC (computerized image analysis)	upregulated in submucosa *n* = 3	Not reported	[31]
		Colon biopsy–affected and non-affected regions	IHC (computerized image analysis)	upregulated in mucosa and infiltrating inflammatory cells; *n* = 30	No significant correlation between disease severity and TRPV1 expression	[32]
		Distal colon biopsy	IHC, qPCR	downregulated mRNA	Not reported	[33]
		Colon biopsy	IHC (manual counting by two observers)	downregulated protein *n* = 26	No significant correlation between clinical features and TRPV1 expression	[34]
UC in remission	TRPV1	Distal colon biopsy	IHC, qPCR	downregulated mRNA	Not reported	[33]
		Colon biopsy	IHC (manual counting by two observers)	downregulated protein *n* = 24	No significant correlation between clinical features and TRPV1 expression	[34]
		Rectosigmoid biopsy	IHC	upregulated in patients with IBS-like symptoms; 3.9-fold increase in median number of TRPV1-immunoreactive fibers (CD and UC combined) *n* = 11	Significant correlation between TRPV1 expression and abdominal pain score	[35]
		Rectosigmoid biopsy	qPCR	No significant difference in mRNA levels between asymptomatic patients and healthy controls *n* = 34	Not reported	[36]

**Table 3:** mRNA expression of TRPV1 and TRPA1 in the animal colon (ISH: in situ hybridization).

mRNA	Location	Method	Model, Animal Species/Strain	Ref
TRPV1	isolated crypts, submucosal and muscle layers of distal, middle and proximal colon	qPCR	intact male Wistar rats	[54]
	upregulated in colonic DRG to the distal colon in DSS-colitis		2.5% DSS-treated C57BL/6 mice	[51]
	unaltered in distal colon, cell type not specified		DSS colitis - male C57BL/6 mice	[33]
	CD4+ T cells		primary cell culture from C57BL/6 spleen	[55]
TRPA1	muscularis externa and mucosa of duodenum, ileum and colon; cell type not specified		intact C57BL/6 mice	[53]
	surface epithelium of middle colon	ISH	intact male Wistar rats	[54]
	isolated crypts, submucosal and muscle layers of distal, middle and proximal colon	qPCR	intact male Wistar rats	[54]
	upregulated in distal colon, cell type not specified		DSS colitis - male C57BL/6 mice	[33]

**Table 4:** Protein expression of TRPV1 and TRPA1 in the animal colon (IHC: immunohistochemistry).

Protein	Location	Method	Model, Animal Species/Strain	Ref
TRPV1	intrinsic sensory neurons of the myenteric plexus-longitudinal muscle of ileum and colon	IHC	intact Sprague-Dawley rats and Dunkin-Hartley guinea pigs of both sexes	[52]
	mucosa, submucosal layers, myenteric plexus and mucosal layer of rectum, distal, transverse and proximal colon		male ddY mice	[50]
	immunopositive neuron fiber density is higher in the distal than the proximal colon		intact and 2.5% DSS-treated C57BL/6 mice colon	[51]
	enteric ganglia, epithelial cells of the distal colon, myenteric and submucosal plexuses, mucosal macrophages, leukocytes		male C57BL/6 mice	[33]
	membrane of resting CD4+ T cells	immunoblotting, flow cytometry, confocal microscopy	primary cell culture from C57BL/6 spleen	[55]
TRPA1	distal colonic epithelial cells, myenteric and submucosal plexuses, interstitial macrophages	IHC	male C57BL/6 mice	[33]
	myenteric and submucosal ganglia; surface epithelial cells of small and large intestines		intact C57BL/6 mice	[53]
	surface epithelium of middle colon		intact male Wistar rats	[54]
	membrane of resting CD4+ T cells	IHC, confocal microscopy	primary cell culture from C57BL/6 spleen	[37]

**Table 5:** Role of TRPV1 and TRPA1 in animal models of colitis (*Trpv1^−/−^, Trpa1^−/−^* gene deleted mice were bred on C57BL/6 background).

Approaches	Results	Animal Strain/Species	Model	Ref
TRPV1 antagonist	*reduces* colitis severity	Sprague-Dawley rats	5% DSS + capsazepine	[56]
		female BALB/c mice	5% DSS + capsazepine/JNJ 10185734	[57]
		Sprague-Dawley rats	TNBS + capsazepine	[58]
		female Wistar rats	TNBS + BCTC	[59]
		*IL10^−/−^Trpv1^−/−^* mice	*IL10^−/−^* -induced spontaneous colitis + SB366791	[55]
TRPV1 agonist	*attenuates* colitis/visceral hyperalgesia	male Sprague-Dawley rats	TNBS + capsaicin	[60]
		male BALB/c mice	DNBS + curcumin	[61]
		male Sprague-Dawley rats	5% DSS + curcumin	[62]
TRPV1 gene deletion	*decreases* colitis	*female Trpv1^−/−^*mice	2% DSS	[63]
		*IL10^−/−^Trpv1^−/−^* mice	*IL10^−/−^*-induced spontaneous colitis	[55]
		male *Trpv1^−/−^*mice	2% DSS	[64]
	*aggravates* colitis	*female Trpv1^−/−^*mice	DNBS	[65]
	*does not affect* colitis severity	*female Trpv1^−/−^*mice	5% DSS	[63]
		*Trpv1^−/−^* mice	TNBS	[66]
		*Trpv1^−/−^* mice	2.5% DSS	[67]
	*protects against chronic pain* during recovery	*Trpv1^−/−^* mice	2.5% DSS	[67]
	*decreases* CD4+ T cell activation and cytokine production	*IL10^−/−^Trpv1^−/−^* mice	*IL10^−/−^*-induced spontaneous colitis	[55]
TRPA1 antagonist	*reduces* colitis severity	C57BL/6 mice	TNBS + HC-030031; DSS + HC-030031	[66]
	*reverses* visceromotor response	female Wistar rats	TNBS/ethanol + TCS-5861528	[59]
TRPA1 gene deletion	*decreases* colitis	Trpa1^−/−^ mice	TNBS, 2% DSS	[66]
		male *Trpa1^−/−^* mice	2% DSS	[64]
	*aggravates* colitis	*male Trpa1^−/−^* mice	2% DSS	[33]
		*IL10^−/−^Trpa1^−/−^* mice	*IL10^−/−^*-induced spontaneous colitis	[37]
	*increases* TRPV1 channel activity in CD4+ T cells, *increases* CD4+ T cell activation and proinflammatory cytokine production	*IL10^−/−^Trpa1^−/−^* mice	*IL10^−/−^*-induced spontaneous colitis	[37]
Capsaicin-induced sensory desensitization	*aggravates* colitis	female BALB/c mice	oxazolone	[68]
		male *Trpv1^−/−^, Trpa1^−/−^* mice	2% DSS	[64]
	*alleviates* colitis	Sprague-Dawley rats	5% DSS	[56]
RTX-denervation	*alleviates* colitis	C57BL/6 mice	TNBS, 2% DSS	[66]
